# Infant feeding for women with an eating disorder: An interpretative phenomenological analysis

**DOI:** 10.1111/mcn.13710

**Published:** 2024-08-20

**Authors:** Dawn Leeming, Samantha Barnsley‐Bridger, Rumaanah Shabir, Sophie Hinsliff, Joyce Marshall

**Affiliations:** ^1^ School of Human and Health Sciences University of Huddersfield Huddersfield UK; ^2^ Faculty of Arts and Social Sciences The Open University Milton Keynes UK

**Keywords:** anorexia, breastfeeding, bulimia, eating disorder, infant feeding, interpretative phenomenological analysis, maternity care

## Abstract

Mothers with eating disorders can face additional challenges with infant feeding, and there is evidence they are likely to cease breastfeeding earlier than intended. However, there is little research exploring this. The present study used interpretative phenomenological analysis to explore the lived experience of infant feeding for mothers suffering from or recovering from an eating disorder. Semistructured interviews were conducted with six women—five who had breastfed and one who formula‐fed. The women experienced two incompatible worlds—motherhood and an eating disorder. Tensions were sometimes resolved by reducing eating disordered behaviour alongside immersion in motherhood. Two participants did not find infant feeding particularly important for their journey into motherhood. Four recounted a positive shift in their relationship to their body through breastfeeding and felt their embodied experience of mothering provided a route out of eating disordered behaviour. However, doubts about their mothering and infant feeding capabilities could be amplified by feeling mistrusted by others and by the relative silence around eating disorders within maternity care services. Respectful dialogue with health care professionals was particularly valued where this occurred. Although long‐term outcomes for the participants are unknown, the study suggests women with a history of eating disorders can form successful breastfeeding relationships and may be motivated to engage in collaborative risk assessment. However, they need support in managing emotional challenges. Training around eating disorders for maternity care professionals is likely to be useful for enhancing confidence in engaging mothers proactively to share concerns about eating, weight and body shape.

## Key messages

- This in‐depth analysis of six women's lived experience illuminates the additional, often hidden, emotional challenges that women with a history of eating disorders can face in early motherhood.
- The findings suggest that becoming a mother, and breastfeeding in particular, can motivate a reduction in eating disordered behaviour and help some women to see their body in a more positive light.
- The women's accounts point to the need for individualised infant feeding advice that addresses concerns about eating, weight and shape and is part of respectful, collaborative discussion between mothers and health care professionals.

## 1 INTRODUCTION

Approximately 5% of pregnant women may meet criteria for a current eating disorder and 15% are likely to have experienced an eating disorder previously (Bye et al., 2021). Therefore, it is important for health professionals working with pregnant and post‐partum women to understand how disordered eating might impact transition to motherhood, especially as the limited research has suggested it may be more difficult for women with eating disorders to enjoy motherhood (Bye et al., 2021; Koubaa et al., 2008).

The term ‘eating disorder’ is applied to a range of behaviours and attitudes towards eating that have a significant impact on health and wellbeing and may be relevant to infant feeding. They include extreme restriction of food intake, uncontrolled binge eating and purging through vomiting, laxatives or excessive exercise, extreme concerns about weight and shape, distorted body perception and feelings of low self‐worth (Waterhous, 2017). Although there are debates about distinctions between different patterns of disordered eating, the most commonly used diagnostic labels for specified eating disorders are anorexia nervosa, bulima nervosa and binge eating disorder (American Psychiatric Association, 2013).

This paper focusses on the way in which eating disorders impact an important aspect of early motherhood—infant feeding. Despite the health benefits of breastfeeding for both mothers and children, population‐wide breastfeeding rates remain of concern, with the lowest rates of continued breastfeeding in high income countries (Victora et al., 2016). To better understand why women may not initiate or continue breastfeeding, Spencer (2008) called for an ontological reorientation of research into breastfeeding to focus on the psychosocial experiences of new mothers, using methods that enable the complexities of their infant feeding experiences and decision‐making to be explored in their social context. Since then, there has been a growth in qualitative analyses of women's experiences of infant feeding (see reviews by Afoakwah et al., 2013; Beggs et al., 2021; Hauck et al., 2021; Lackshman et al., 2009; Morns et al., 2021; Regan & Ball, 2013). However, there has been limited attention to the infant feeding experiences of women with a history of struggling with eating and weight concerns.

Even for mothers without eating disorders, infant feeding experiences are complex and multi‐layered. Within western societies, the meaning of breastfeeding is contested. Conflicting messages include public health campaigns interpreted as ‘breast is best’, alongside cultural messages around the sexualisation of breasts. Significant others within women's social networks are sometimes sceptical that breast milk will provide sufficient nutrition and view formula as an equivalent alternative (Leeming et al., 2022). Therefore, on the one hand, breastfeeding may be viewed as symbolic of motherhood—an important part of bonding and mothers' developing bodily relationship with their baby, providing a sense of personal achievement and intimate connection with their baby for many (Afoakwah et al., 2013). On the other hand, breastfeeding may be experienced as an unproductive time (Leeming et al., 2013), a risky activity needing medical supervision (Regan & Ball, 2013) and involving disgusting bodily fluids (Dowling et al., 2012) or a challenge to bodily autonomy and control (Afoakwah et al., 2013). Some mothers can find breastfeeding aversive (Morns et al., 2021). Others may experience shame, guilt or embarrassment related to feeding in front of others or in response to difficulties establishing breastfeeding or formula feeding (Hauck et al., 2021; Leeming et al., 2022; Thomson et al., 2015). Although most mothers begin breastfeeding, many introduce formula feeding and do not continue breastfeeding as long as they had intended to nor as long as WHO recommendations (e.g., McAndrew et al., 2012). The meanings attached to formula feeding can range from relief at finding a pragmatic solution, pleasure that others can be involved in the feeding (and therefore bonding) process (Lee, 2007) to disappointment, guilt, failure and shame (Leeming et al., 2022; Thomson et al., 2015).

New mothers navigate these contradictory meanings at a time of significant transition. Becoming a mother can be experienced as a loss of control and a renegotiation of self‐concept (Bollen, 2015). Women often report dissatisfaction with their post‐natal bodies, regardless of whether they have an eating disorder (Coker & Abraham, 2015), and breastfeeding women in particular need to construct meaning about a new body shape with larger, differently shaped breasts. It is reasonable to assume that navigating these changes, decisions and related meanings might be more difficult for women who already have significant concerns about body image and weight gain. Moreover, disordered eating is often associated with perfectionism, low self‐esteem and significant feelings of shame (Nechita et al., 2021; Puttevils et al., 2019), where self‐worth can be bound by shape and weight and a sense of achievement and control produced by self‐starvation (American Psychiatric Association, 2013). Some women with eating disorders may be less well prepared for motherhood as rates of unplanned pregnancy seem higher, possibly because they believe they cannot conceive (Bulik et al., 2010; Taborelli et al., 2016).

Bye, Martini and Micali's (2021) review concluded that pregnancy and the post‐natal period could indeed be challenging for women with a current or previous eating disorder. They are particularly likely to experience post‐natal dissatisfaction with their body and difficulties adjusting to motherhood (Koubaa et al., 2008; Taborelli et al., 2016) and to report infant feeding difficulties (Micali et al., 2009). They are also more likely to experience post‐natal anxiety and depression (Bye et al., 2021). Although some women may initially find motherhood helps to overcome their eating disorder, others find these two aspects of their lives conflict (Fogarty et al., 2018; Tierney et al., 2011), and there is evidence that difficulties around eating often return (Bye et al., 2021).

Research findings regarding the relationship between breastfeeding rates and eating disorders have been mixed. Some studies demonstrate higher rates of breastfeeding (Micali et al., 2009), while others show that although mothers with eating disorders may have similar breastfeeding initiation rates to other mothers, earlier cessation is more likely (Torgersen et al., 2010). A recent systematic review on the impact of maternal eating disorders on breastfeeding practices (Kaß et al., 2021) confirmed that some women with eating disorders breastfed for a shorter duration, while reporting more emotional problems relating to breastfeeding, compared to healthy controls, and recommended further qualitative investigation of this. The limited qualitative research available suggests that women can feel torn between prioritising their child's welfare or engaging in disordered eating and can easily feel undermined by breastfeeding difficulties (Tierney et al., 2011). Anxiety around infant feeding decisions may relate to concerns about weight loss following birth and whether breastfeeding might promote this or would curtail disordered eating behaviour (Fogarty et al., 2018; Larsson & Andersson‐Ellström, 2003; Stapleton et al., 2008; Torgersen et al., 2010). Slightly increased concerns about infant weight have also been noted for women with current and previous eating disorders (Martini et al., 2019). One of the few qualitative studies with a particular focus on infant feeding and eating disorders noted that women felt partners had urged them to formula feed because they did not trust them to eat sufficiently well to breastfeed, though partners were not interviewed (Stapleton et al., 2008). An earlier study suggested some women with a history of both anorexia and bulimia may find breastfeeding intrusive (Morgan et al., 1999), though a survey reported no differences in ratings of positive breastfeeding experiences between women with and without an eating disorder (Larsson & Andersson‐Ellström, 2003). However, there has been little sustained in‐depth attention to experiences and understandings of infant feeding for this group of women (Kaß et al., 2021; Martini et al., 2019).

The aim of this study was to explore the lived experience of navigating infant feeding while managing or recovering from disordered eating. This can help to inform support so it is attuned to the meanings women attach to these experiences, the challenges they face and the ways in which they have been able to address these challenges.

## 2 METHODS

### 2.1 Design and theoretical framework

Interpretive phenomenological analysis (IPA) (Smith et al., 2009) was adopted as a method of enquiry and data analysis. IPA aims to explore lived experience as it appears in consciousness but, while seeking to ‘give voice’ to participants, attempts to make sense of this experience through interpretation. It prioritises in‐depth analysis of small groups of participants and close reading of individual accounts (rather than aiming for data saturation) but also explores commonalities and divergence in experience and meanings (Nizza et al., 2021). Drawing on Heidegger's phenomenology and hermeneutics, IPA is concerned with both subjective experience and the meaning of this for participants, as interpreted by the researcher (Smith et al., 2009, 2022).

### 2.2 Participants

Mothers with eating disorders who were willing to be interviewed about experiences of infant feeding were recruited via an advert posted, with permission, on the website of BEAT (Beat Eating Disorders), a UK charity which supports individuals affected by eating disorders. Six responded and agreed to take part. Given IPA's emphasis on in depth analysis of small samples and the quality of the six participants' data, the data set was deemed sufficient. Eligibility criteria were that participants were mothers with at least one child under 3 years of age and had a current or recent eating disorder diagnosis. To retain a focus on the women's own meanings, we did not assess participants further in relation to diagnostic criteria. However, checking that they had received a diagnosis confirmed that health care professionals had considered their difficulties significant. Five of the six women recruited breastfed their babies. All were between the ages of 31–39, resident in the United Kingdom and self‐identified as women. Additional participant information is provided in Table 1.

**Table 1:** Participant information.

**Name**	Elle^a^	Emma	Jess	Sofia	Katy	Sarah
**Age**	37	39	36	35	31	33
**Reported diagnosis** b	Anorexia (in recovery)	Anorexia (current diagnosis, but also viewed self as in recovery)	Bulimia (current diagnosis)	Anorexia (purging type—Current diagnosis)	Anorexia (in recovery)	Bulimia (in recovery)
**Vocation**	Peer support worker^c^	Health professional	Engineer	Peer support worker^c^	Educator	Business owner
**Feeding method**	Breastfeeding + expressing	Exclusively breastfeeding	Breastfeeding + expressing	Exclusively breastfeeding	Exclusively breastfeeding	Formula feeding
**Number of children**	Two	One (pregnant with second)	One	Three	Two	Two
**Age of youngest at interview**	6 months	18 months	3 years	12 months	21 months	3 years
**Weaning age for youngest**	6 months+	11 months	10 months	12 months+	21 months+	N/A
**Ethnicity**	White British	White British	White European	White British	White British	Unknown

^a^ Pseudonyms are used throughout.

^b^ Diagnosis as reported by participants, including whether this was current and/or whether they saw themselves as in recovery.

^c^ Voluntary role.

### 2.3 Data collection

A semistructured interview schedule maintained the focus on infant feeding in the context of experiencing or recovering from an eating disorder, while exploring the nuances of idiosyncratic lived experience. All participants opted for telephone rather than face‐to‐face interviews. The interviews were conducted by one of the authors, a graduate masters‐level psychologist, under the supervision of two authors with backgrounds in Clinical Psychology and Midwifery. Interviews began by establishing the women's chosen methods of infant feeding, followed by open questions, in a conversational style, exploring feeding experiences during the first 12 months post‐partum and beyond, including positive aspects of infant feeding and any challenges, feelings about becoming a mother, interactions with others related to infant feeding (including health professionals) and the sense the women made of the relationship between their eating issues, infant feeding and mothering. The interviews ranged between 43 min to an hour and were audio recorded and transcribed in full.

### 2.4 Data analysis

The transcribed interviews were analysed using the original version of IPA (Smith et al., 2009). After repeated re‐readings to gain close familiarity with the transcripts, pertinent themes and patterns arising from each individual account were identified inductively by the interviewing author, before looking for commonalities across the set of interviews, in discussion with the two other team members who had supervised the interviewing. The initial analysis was then reviewed, revised and elaborated by two additional team members who had not been involved in the interviewing and who had qualifications in Counselling, Psychology and Midwifery. Examination of emerging individual themes and exploration of the connections between participants' shared experiences led to mapping out three master themes with subthemes and one overarching theme. This thematic structure articulated what were agreed by the team to be the most pertinent issues across the participants, with the overarching theme (*Incompatible Worlds: Motherhood vs. my Eating Disordered Self)* developed as an attempt to capture what appeared to be the most salient aspect of the lived experience of infant feeding with a history of disordered eating.

### 2.5 Validity and rigour

In keeping with recommendations for quality within IPA (Nizza et al., 2021), we remained close to the experiences of the participants, avoiding wider claims and theoretical assumptions and aiming for an experiential account focused on meaning making. Throughout analysis, attention was paid to convergence and divergence. We maintained a reflexive approach by frequent rereading, the keeping of a reflexive diary by the interviewing author and mutual discussion, aided by our varied perspectives and backgrounds. While all authors are mothers and had prior experience of qualitative analysis, two of us are midwives, two have worked with women with eating disorders in counselling/mental health contexts, and we have different ethnic and cultural backgrounds. IPA requires researchers to be mindful of pre‐understandings, recognising that bracketing of these can never be fully achieved (Smith et al., 2022). It is worth noting that all of us recognised a prior expectation that women with eating disorders might face some difficult emotions in relation to infant feeding, and it was important to ensure, through reflection and discussion, that this expectation was questioned and did not dominate our analysis of the women's accounts.

### 2.6 Ethical statement

The research was approved by the School of Human and Health Sciences Research Ethics Panel of the University of Huddersfield, UK. Before interview, all participants provided informed consent and were briefed on their right to withdraw, their anonymity, their well‐being and the limitations of confidentiality. All participants are anonymised throughout, and pseudonyms are used. We were mindful of the sensitivity of the interviews, and it was made clear to participants that all perspectives and experiences were valued, that they were not obliged to answer questions they did not wish to and could stop the interview at any point. At the end of the interview, they were reminded of the helplines and resources available via BEAT and health services, and this was reiterated in a follow‐up debrief email.

## 3 RESULTS

Table 2 below indicates the three master themes and subthemes that were developed from the interpretative phenomenological analysis of the participants' lived experience. Together these themes contributed to the overarching theme of ‘Incompatible worlds’. This overarching theme is discussed first, followed by discussion of each contributory master theme in turn.

**Table 2:** Themes from interpretative phenomenological analysis.

**Overarching theme**	Incompatible worlds: Motherhood vs. my eating disordered self		
**Master themes**	1. The challenges of negotiating infant feeding and an eating disorder	2. Escaping from an eating disorder via mothering	3. The importance of trusting myself and feeling trust and validation from others
**Subthemes**	An anxiety‐provoking body Negotiating a loss of control The strain of a dual focus	Breastfeeding as therapeutic and affirming Caring for myself to care for baby A new purpose and direction	Doubting abilities and bodies Under surveillance Helpless in the face of silence and taboo Feeling respected and validated

### 3.1 Overarching theme: Incompatible worlds: Motherhood versus my eating disordered self

Although one participant Jess, who was still struggling with bulimia, spoke about being “hell bent on breastfeeding” because this would enable her to lose weight, more commonly participants (including Jess) spoke as if the two identities—mother and woman with an eating disorder—were difficult to hold simultaneously and represented incompatible worlds. Five of the six participants had breastfed, and several linked their contradictory mother/eating disordered identities explicitly to breastfeeding:

When I'm with my children and feeding them [exclusive breastfeeding] I'm not, it might not make sense, but I'm not anorexic—Sofia

Elle talked about these as two irreconcilable ways of living life:

… if you're struggling quite a lot [with anorexia] then it would be very difficult to have this person need you so much for their nutrition, and also invading that space and kind of interfering in your ability to actually be anorexic.

While the opposing tensions of breastfeeding and motherhood could be troubling, several women talked about motherhood giving them a new focus and purpose that *replaced* their focus on the eating disorder. For Emma, this had been transformative, despite still seeing her diagnosis as having some currency:

You think that the anorexia is always going to be there and you're always going to be battling [the difficulties] all of the time…having a baby has completely turned my life upside down, in so many ways for the better and my life is now my little boy and my husband and this other little one…why would I want to jeopardise that by having anorexia back?

Katy and Elle, who were in recovery, and Emma and Sofia, who viewed their diagnosis as current, spoke of finding a higher importance in motherhood which diluted their focus on their eating disorder and themselves. Breastfeeding had played an important part in this for them. Jess and Sarah had not felt that infant feeding was a particularly important part of their mothering journey, though also spoke of the role motherhood had played in reducing eating disordered behaviour. There was a sense of agency within some of the women's accounts at times—as if they could choose motherhood or an eating disorder. However, at the same time, they spoke about their disordered eating as a lived experience of something they ‘battled’ or ‘struggled’ against, with limited control. Motherhood provided a different identity and life path and could be experienced as protecting against disordered eating, though being torn in different directions was difficult. The following themes (illustrated in Table 2 above) discuss key aspects of the women's experience of navigating these conflicting worlds: the experience of tensions and ongoing negotiation between two identities (Theme 1), the way in which motherhood could provide some means to escape an eating disorder, particularly through breastfeeding (Theme 2), and, given their eating disordered history, the difficulties of trusting in their mothering and feeding abilities and feeling trusted by others (Theme 3).

### 3.2 Master theme 1: Challenges of negotiating infant feeding and an eating disorder

To continue their infant feeding journey, the mothers faced a conflict between holding on to or releasing concerns about eating and their bodies.

*An anxiety‐provoking body:* Concern with body shape and weight—that their bodies had not returned to ‘normal’—was a challenge for most. Jess, whose diagnosis of bulimia was current, had been particularly concerned about her shape and weight since pregnancy and felt alienated from her breastfeeding body:

The way I looked was stressful, big breasts, very round, I wanted to be myself and that wasn't me, that wasn't how I looked before.

She was the only participant to explicitly cite weight loss as a factor in deciding to breastfeed her baby. Katy referred to the connection between breastfeeding and weight loss, though had in fact found that breastfeeding involved weight gain. She noted the importance of ‘how you feel about your body’ in enabling women to breastfeed and how, for her, concerns about increased weight would come to the fore when aware of the potential of others' gaze on her body:

It [weight gain] would make me feel more aware of when I was feeding in public, made me feel a bit more conscious about what my body shape and size was.

At times when she felt low or under pressure, she had found the weight gain particularly difficult to tolerate, becoming critical of herself and her body and developing strategies for dealing with this such as eating less and walking.

*Negotiating a loss of control:* Emma and Elle struggled with a sense of losing control. This not only destabilised their inner worlds but had a resonance with eating disordered thoughts and behaviours:

Suddenly this little person had arrived and having no control over my life and I was just feeling quite unsure about myself, not feeling like I wanted to engage with people or go out and do things, so there was a little bit of the feelings from when I suffered with anorexia creeping back in—Emma

Emma's quote shows how the lack of control and uncertainty associated with early motherhood was potentially triggering for her. As noted in the overarching theme (above), Elle spoke about how breastfeeding could be experienced as reducing control over one's body—‘invading that space’ and ‘interfering’ with a mother's choices. Jess made this explicit:

I felt like my body wasn't my own, like I had no control over it.

*The strain of a dual focus*: Mothering and disordered eating were often experienced as activities which competed for central focus, requiring additional resources. Jess described feeling that she no longer had the capacity for eating disordered behaviour, even though she still referred to her bulimia as active:

…an eating disorder takes lots of energy and lots of planning, you eat, you need to then find time to get rid of it, so throw it up or burn it, but other things become more important and there's just no time for it. So that's one reason, it takes up a lot of energy because it's not just mental energy but physical energy, erm, I didn't feel like I had it.

Jess had found pregnancy and breastfeeding particularly difficult to the point where she questioned whether having any more children would be worth the bodily sacrifice. She felt she had had very little support from those around her in her decision to breastfeed and to maintain a breastfeeding relationship with her baby.

Similarly, Sarah, who formula‐fed, focussed on how ‘time consuming’ bulimia was and the difficulty of combining this with first time motherhood. Katy also experienced her time as stretched by a dual focus. She noted the difficulty of lengthy breastfeeds which meant she ‘never had any time for myself’, such as time to take care of her own eating in a way that was important to her—to prepare food that was ‘nice and healthy’. However, despite finding these pressures distressing (‘I remember sobbing quite a lot’), she valued the way in which the demands of motherhood could provide distraction from her concerns around weight and eating, as discussed more fully in the next theme:

It's [the eating disorder] still there it's not there as much, but I think it's because I'm so busy I don't really get time to think about it as much as I would have done before I had children.

There was a sense that time constraints were temporary, though, and some of the participants questioned whether this would prevent further eating‐disordered behaviours in the future. However, for the present, Katy and others spoke as if they had little choice and the dual focus shifted to a focus on motherhood. Katy added:

I just had to sort of get on with it really, just try and, I don't know a bit mind over matter and trying not to focus on me and try and focus on the babies

### 3.3 Master theme 2: Escaping from an eating disorder via mothering

This theme explores more fully how the embodied experience of motherhood could offer an alternative to and respite from disordered eating, at least temporarily.

*Breastfeeding as therapeutic and affirming:* Four of the five breastfeeding women explained how breastfeeding contributed to feeling more positive about their body and its capabilities and their role as a mother:

My issues with the eating disorder were more prominent when I was pregnant, … but since his birth the breastfeeding has helped, with me trusting my own body and seeing it can provide nutrition for my baby, seeing what it can do can actually be a positive thing rather than something to battle with—Sofia

This reflected not just pride in the achievement of providing good nutrition for her baby but also a new and positive relationship to her body, as Sofia continued to struggle with anorexia. Katy who had previously also felt negatively about her body expressed similar sentiments, adding that breastfeeding provided safety as well as achievement:

I'd say it's sort of like a little protective bubble that's a distraction or a comfort I'd say, to know that I'm still doing something right.

Later, in the interview, she explained how she too had come to see and experience her body in a new way through the act of feeding:

Breastfeeding has helped … me see my body in a different way, that actually my body is good for what it does and it's brilliant and actually I should cut it some slack every now and again [laughs] it's done a very good job and I shouldn't be so mean to it sometimes. It's helped me produce two beautiful children and erm it's helped me look after them and feed them and nurture them and I've sort of grown to love my body in a different way.

For many of the women, feelings of satisfaction and achievement in their breastfeeding journey smoothed the transition to a new identity, where they could actively choose to focus on family life and parenting as an alternative to their previous anorexic/bulimic identity:

[Breastfeeding is] kind of a calming thing and quite grounding…It's very much about the bond, the intimacy and sense of connection between the baby and also the connectedness within yourself, it's actually really helpful in that way, to distract from, like anorexia—Sofia

*Caring for myself to care for baby:* The sense of their body as productive rather than anxiety provoking made it seem acceptable to look after their physical selves:

Nursing [breastfeeding] her helps me, that I'm doing something right and actually I can do it, I'm okay and I need to look after myself to look after her—Katy

I just had to focus on my son really…the life of this little baby is in my hands now and I need to look after myself properly to be able to look after him—Emma

Some of this sounded functional—a recognition that their bodies, where breastfeeding, needed to be nurtured to nurture. However, as suggested by Katy's quote and her earlier comment that she should ‘cut it [my body] some slack’, they were now allowing themselves to care for and be kind to their bodies in a way that had not seemed acceptable previously.

*A new purpose and direction:* A couple of the participants felt they had incorporated the energy usually reserved for eating disordered behaviours into mothering and infant feeding. They made sense of this as repurposing a strong will, a focus for goals and a need for control, directing this towards infant feeding, particularly breastfeeding:

I think most people who have anorexia have quite strong wills and they're determined, and they focus on goals and things like that, and because breastfeeding has always been you know, this “I want to have children and I want to breastfeed”, I want to you know, achieve my goals and that makes me feel really positive—Elle

Not all the women valued breastfeeding. Sarah did not breastfeed, and Jess spoke of feeling relieved when she stopped breastfeeding. However, they still spoke about other situations where immersion in mothering provided a new purpose. Sarah focussed on protecting her children from the effects of an eating disorder or from developing similar difficulties and expressed a desire to set a good example:

It wasn't until I heard what my older daughter said about me being sick until a light just switched on and I was like no that's it now, I need to get through this.

Motivated by her daughter's awareness, she was able to gain control over her eating disordered behaviour through therapy, before the birth of her subsequent child.

Thus, the participants could feel some resolution of the tensions between an eating disorder and motherhood by pulling away from disordered eating. Elle stated particularly strongly how she felt becoming a mother had rescued her from her eating disorder:

I was really quite poorly, you know I'd go up and down…. So, in some ways, probably having babies has saved my life, it's given me something to live for, you know rather than living and having anorexia.

### 3.4 Master theme 3: The importance of trusting myself and feeling trust and validation from others

Despite these accounts of embracing motherhood, the women sometimes doubted their mothering and feeding abilities. This could be exacerbated by feeling that others also mistrusted their mothering and feeding because of their history of disordered eating or that professionals would mistrust them if they knew the full extent of their eating disorder. Therefore, support and validation from health care professionals was highly valued where this was present, but silence around eating disorders within relationships with professionals made it difficult to feel fully validated as a mother with an eating disorder.

*Doubting abilities and bodies:* Katy explained how self‐critical thoughts could undermine her confidence in herself:

Without even realising, I sometimes end up cutting back on food or my appetite goes or I'm not eating much, and then you end up with the little voices in your head sort of erm, criticising yourself or how you look and how you feel.

The women were at various stages of recovery and most experienced residual thoughts and feelings that they saw as part of the eating disorder, which led them to doubt their abilities and bodies. Sofia, who was still struggling with purging, highlighted particularly strongly how this left her second guessing herself:

I just had a lot of self‐doubt and worry that the eating disorder is trying to trick me and that I wasn't looking after myself…I kind of distrusted myself and what my motivations were.

Most of the women who breastfed also had some concern with supply and quality of milk, as indicated by Jess:

That was the main concern I think, that he's not getting enough nourishment from me.

While such worries may not be unusual for breastfeeding mothers, concerns about adequate calorie intake and trust in their body's capacity to produce what was needed for their infant seemed particularly noticeable for this group.

*Under surveillance:* Alongside this self‐doubt, many women felt as if others also monitored and doubted their ability to feed their baby, whether that was health professionals, family or both. For example, Sofia explained how breastfeeding her second child raised too many concerns for some health professionals, and they were reluctant to trust her with the decision:

Breastfeeding was quite a concern for the health professionals. …I really had to fight to breastfeed him……I felt like they didn't trust me and weren't respecting my decision about me and my children and … my own beliefs about motherhood.

This very visible doubt about her ability to feed her baby left her feeling undermined as a mother and unsupported:

It would have been nice if I suppose, people believe in you rather than thinking you can't do it.

*Helpless in the face of silence and taboo:* Although she wanted health professionals to validate her as a mother, Sofia was hesitant to fully discuss her eating disorder with them. She perceived that someone known to be struggling with an eating disorder would not be trusted. Her diagnosis was current, but seeking help felt very risky and her need for support taboo:

If I asked for support for me, people would then think I was not looking after my children properly, so it kind of made things difficult.

Sarah similarly felt unable to ask for specific support due to assumed stigma about her eating disorder, implying that it would be embarrassing to mention this to health professionals:

It's not really something you want to disclose to people all the time, if they say ‘oh, have you got any problems?’ It's not something you want to tell and say ‘yeah, I suffer from bulimia’.

Therefore, the silence about their eating disorder meant that it was difficult for them to receive confirmation that they were trusted as mothers in spite of their difficulties. Although Katy did disclose her eating disorder, the way she spoke about this indicated it had taken a particular effort to disclose this to a professional ‘because I thought if I don't [disclose] then I don't want to get all secretive and getting silly about it’ (Katy).

Across the interviews there often seemed to be an assumption that, ultimately, being a mother with an eating disorder was something to be managed alone, without clear guidance from others on which to build confidence. This was not just due to a sense of shame or embarrassment about disclosure but also the perception that professionals lacked relevant knowledge. Most spoke about the difficulties they had experienced in accessing advice about nutrition appropriate to their situation, with some having been told by health professionals that there was little guidance available for breastfeeding mothers with eating disorders. Sofia described how this silence not only left her without the information she needed to inform her decisions but also feeling disempowered in her interactions with health care professionals who had not wanted her to breastfeed:

I didn't have faith in what [the health professionals] were saying. But I couldn't say, I had nothing to kind of go on or guide me because all the guidelines [on breastfeeding] are for people who don't have eating disorders.… if purging would have any effect or just things like that, but it's not there.

This made it difficult for her to engage and collaborate with professionals in weighing up the risks and benefits of breastfeeding in a mutually trusting way. Instead, her inner experience was of being in opposition, distrusted, but helpless to counter this.

Similarly, Katy described feeling helpless and lacking in knowledge and confidence about nutrition due to the absence of advice tailored to her concerns. Information saying: ‘you should be having this, and you should be eating this’ seemed ‘just really overwhelming’. When told to eat more protein:

I didn't know what to do, what to do with that amount of information, what is a better amount of protein to eat? What form can it be in? What's the best way to get protein but for it not to be calorific?

She felt that it was not just more focussed guidance that she needed but also help to deal with the emotional implications of the guidance she did have access to:

You sort of get ‘you should be having so much of this and so much of that’ well to me that's a huge amount of food to consume in one day erm so I don't know just a little bit of support or advice or guidance or somewhere you could go to talk to just to help you manage those thoughts and feelings and process it all.

So, silence around eating disorders undermined some women's confidence in their breastfeeding, leaving them alone to deal with related anxieties and uncertainties, while a sense of taboo and fear of being seen as a risk to their children could hold them back from the more genuinely collaborative discussion needed to enhance professionals' trust in their mothering. This was in spite of some mothers' apparent willingness to discuss nutrition in detail. However, as Katy's comments above imply, it is taken for granted that it would not be appropriate to share ‘those [eating disorder related] thoughts and feelings’ with maternity care professionals, though it is unclear why not.

*Feeling respected and validated:* Despite often experiencing silence or an absence of information, many women did find some support that recognised their individual concerns around eating and weight, did not necessarily see these as incompatible with breastfeeding, and demonstrated trust in their mothering. Sofia's experience of support with her third child had been ‘much more of a respectful dialogue with health professionals’. This contrasted with the distrust, silence or unhelpful instruction experienced previously. For Elle, supportive respectful dialogue arose through midwives and health visitors making links with mental health professionals and together supporting her to meet her goals:

We kind of figured that [breastfeeding] was something that was really important to me…so yeah, we had a care plan and they made sure…I had enough support…and that I was aware of all the places I could get support from once I came home.

Several of the women spoke about encouragement from families and general breastfeeding services and began developing trust in their mothering and infant feeding, mitigating some of the earlier doubts. When discussing the value of group therapy, Sarah particularly highlighted the value of informed supporters who recognised the silent, secret struggle to mother well with an eating disorder:

It's a dark place to be in, it's a secretive place, people think you're alright and you're not really, and to just have that bit of moral support from people that know what they're talking about, I think that can make all the difference.

## 4 DISCUSSION

The mothers in this study experienced a tension between the perceived demands of motherhood and those of an eating disorder. This mirrors previous research (see review by Fogarty et al., 2018). However, the current findings present a more optimistic picture of the post‐natal period than previous research suggesting that, despite the tensions and challenges, the demands of motherhood could be a valuable part of recovery or partial recovery from an eating disorder. For several of our participants, breastfeeding helped affirm their mothering identity, as is the case for many women without an eating disorder (Afoakwah et al., 2013). Breastfeeding could also foster a more positive relationship with their bodies and a new imperative to look after themselves—a shift which the research literature on eating disorders has previously related to pregnancy, rather than the post‐natal period (Fogarty et al., 2018). To some extent, all participants described moving towards a mothering identity and away from eating disorder activities, as early motherhood and infant feeding could be an all‐encompassing and intense time of refocus and distraction from prior states of being.

Despite this positive shift, many also experienced troubling emotions and thoughts. Becoming a mother can be emotionally complex, regardless of an eating disorder. It is not uncommon for women to experience initial concerns about breastfeeding, including whether their infant is feeding adequately, and their milk supply is sufficient, alongside concerns about exposure, public feeding, failure, bodily autonomy and managing their own and others' discomfort (Afoakwah et al., 2013; Beggs et al., 2021; Hauck et al., 2021; Leeming et al., 2022). However, for some of the present mothers, such challenges were imbibed with additional meaning. Worry around milk insufficiency arose in the context of prior hostility towards their bodies and often seemed overshadowed by the post‐natal concerns about body shape and weight that have been noted in previous research with this group of women (Fogarty et al., 2018; Koubaa et al., 2008; Taborelli et al., 2016). These concerns could add to their self‐consciousness around public feeding. Moreover, loss of autonomy and the need to manage feeling out of control was uncomfortably reminiscent of eating disordered thinking. Women also developed concerns about the impact of their eating disorders on their children.

Several spoke at times as if they were torn between two identities and pulled in two directions—a woman with an eating disorder or a mother. Researchers have previously noted the burden on breastfeeding women of navigating and transitioning between different norms for how the body is used—being faced with a new world where their body is used for nurturing an infant, while attuned to the old world of bodily autonomy to which women may return. This has been discussed using the concept of liminality (e.g., Dowling & Pontin, 2017; Mahon‐Daly & Andrews, 2002) to highlight how women may feel out of place and uncertain, ‘betwixt and between’ competing demands (Dowling & Pontin, 2017). The current findings suggest this analysis can be extended for mothers with a history of an eating disorder. They may experience an additional layer of uncertainty as breastfeeding motherhood requires a stark departure from the old world of disordered eating with its primary focus on bodily denial. Emotions may shift depending on whether their experiences are appraised through the lens of the old world (prioritising prevention of weight gain) or the lens of the new world of motherhood (prioritising adequate nutrition). Our participants seemed acutely aware of the incompatibility of these appraisals but could sometimes be drawn to both. However, despite the tension and uncertainty of this phase, many of the participants felt that their relationship to their body and their identity changed for the better through their engagement with the process of early mothering.

The importance to the participants of individualised guidance and support and positive, collaborative relationships with professionals who had confidence in their mothering and infant feeding mirrors findings for mothers without eating disorders (Leeming et al., 2022; Schmied et al., 2011). Such validation from maternity care professionals may have been particularly important for this group of mothers, given that they alluded at times to feelings of embarrassment, shame or failure. This is not unexpected as eating disorders are often associated with feelings of shame (Nechita et al., 2021), while mothering is one of the most common sources of shame for women (Brown, 2006) and can include feelings of failure or exposure related to infant feeding (Thomson et al., 2015). This points to the importance of professionals working with women to support shame resilience through acceptance, empathy and nonjudgemental discussion (Leeming, 2018). As Dolezal and Gibson (2022) argue, shame‐sensitive practice in health care contexts means providing an environment where it feels safe to disclose shaming concerns, such as disordered eating as a mother, and where these concerns are then discussed directly, to undermine the legitimacy of the idea that these issues are shaming. However, previous UK research with midwives and health visitors found that difficulties with disordered eating were not often mentioned in practice, partly because they felt they had limited training in this area, and hence low confidence addressing or even recognising eating disorders (Bye et al., 2018). Although we did not observe consultations with professionals, our participants also felt they experienced a lack of professional engagement with their eating‐disorder‐related concerns. Moreover, the findings illustrate how mothers too may feel compelled to continue a conspiracy of silence around eating disorders, as if concerns about disordered eating are incompatible with being a good mother.

This research points to the value of further training for maternity care professionals to destigmatise and improve understanding of eating disorders. Within the United Kingdom, some progress has been made in that health professionals increasingly initiate discussions about emotional well‐being during pregnancy and in the post‐natal period, not least because MBRRACE‐UK continue to identify mental health conditions as a leading cause of maternal death (Knight et al., 2023). Guidance on managing perinatal mental health refers explicitly to eating disorders, amongst other conditions, and emphasises that midwives should work within multi‐disciplinary teams to support mothers (National Institute for Health and Care Excellence NICE, 2020; Royal College of Psychiatrists, 2021). Nevertheless, in a systematic review of services users' experiences of accessing perinatal mental health support, Sambrook Smith et al. (2019) noted multiple, complex barriers to support that correspond to those mentioned here, including stigma and lack of knowledge amongst health care professionals.

The current findings also suggest it would be useful to raise awareness that some mothers with a history of an eating disorder (though not necessarily all) may wish to engage collaboratively in discussion and management of risk, even if they are still struggling with issues related to eating and body image. The study highlights that women who struggle with disordered eating and concerns around weight and shape are not passive ‘victims’ of an eating disorder but can be mothers with agency, trying to manage risks to their parenting as well as they can and demonstrating resilience in the face of difficult emotional experiences, others' doubts and limited information and support. Despite previous research having suggested women with eating disorders are more likely to experience infant feeding difficulties (Kaß et al., 2021; Micali et al., 2009), a key finding from the current study that has not previously received much attention is that some women can develop a good breastfeeding relationship with their baby in the aftermath of an eating disorder.

Despite the positive findings about the potential for motherhood to support recovery, it is important to recognise that the present study included only mothers' perspectives, captured in a single telephone interview. We cannot claim that all participants gave full and transparent accounts of the extent of their difficulties around food and body shape. There is still considerable stigma around eating disorders, even within health care contexts (Bye et al., 2018; Fogarty et al., 2018), and it would be naïve to assume that women will divulge the full extent of their difficulties to an unknown researcher. The health care professionals involved may have perceived risks differently, given that research suggests that eating disorders can have a negative impact on children's development (Martini et al., 2020). However, it is also worth noting that several of the current participants acknowledged within the interview that they agreed with some of the health care professionals' concerns, though had felt unable to discuss these openly with professionals due to embarrassment. This suggests that collaborative discussion may be possible if women are asked directly about issues related to their eating disorder.

Study participants were between 31 and 39 years at the time of the interview and many had struggled with, and sought help for, disordered eating for some time. Half regarded themselves as in recovery. Therefore, the experiences of some participants may not be applicable to a younger group with a shorter period of disordered eating. It is also important to note that participants' ages and occupational and educational histories made them statistically more likely to breastfeed (McAndrew et al., 2012). Five participants were white, with one of unknown ethnicity, and we therefore also need to be cautious in transferring these findings to women of other ethnic backgrounds.

It is unknown whether the participants experienced lasting changes in their lived experience of their bodies and themselves. Previous research suggests that disordered eating can return after the initial post‐partum period (Bye et al., 2021; Fogarty et al., 2018), though all participants were at least 6 months post‐partum. However, some of the breastfeeding women expressed uncertainty about weaning and whether the loss of a breastfeeding relationship would mean the loss of factors important for their recovery. Two mothers did not attribute the shift from eating disordered self to mother to infant feeding. Rather, they felt that their relationship with their children developed as their children became more interactive.

## 5 CONCLUSIONS

Despite the caveats above, the findings point to the importance of making eating disorders more visible in maternity services and staff training, facilitating disclosure by mothers and enhancing professional confidence to respond to these disclosures in a way that builds rather than undermines relationships. Unfortunately, there is little research on best practice in this area (Bye et al., 2018). This is of additional concern given that lower levels of concern around eating and weight are common amongst western women (Waterhous, 2017), even if they do not meet the diagnostic criteria for an eating disorder. For all these reasons, it is important to develop this field of research further and also to encourage maternity care professionals to engage in conversations about eating and weight concerns with all women.

## AUTHOR CONTRIBUTIONS

The study was designed by Rumaanah Shabir, Dawn Leeming and Joyce Marshall. Data were collected by Rumaanah Shabir. All authors contributed to analysis and writing, though Rumaanah Shabir took the lead on initial analysis and Samantha Barnsley‐Bridger and Dawn Leeming took the lead on writing. All authors have read and approved the final manuscript.

## CONFLICT OF INTEREST STATEMENT

The authors declare no conflict of interest.

## ETHICS STATEMENT

The study was approved by the School Research Ethics Panel of the School of Human and Health Sciences at the University of Huddersfield.

## Data Availability

Research data are not shared.
